# Multidisciplinary Approach in Management of Currarino Syndrome in a Female Infant: A Case Report on Rare Congenital Triad and Focused Literature Review

**DOI:** 10.1002/ccr3.73257

**Published:** 2026-07-28

**Authors:** Ferdinand Medard Shilikale, Cui Mingyu, Liu Guihai, Rajabu Athumani Bakari, Jiao Chenwei

**Affiliations:** ^1^ Department of Pediatric Surgery Shandong Provincial Hospital Affiliated to Shandong First Medical University Jinan Shandong China; ^2^ Pediatric Surgery Unit, Department of Surgery Muhimbili National Hospital Dar es Salaam Tanzania

**Keywords:** anorectal malformation, Currarino syndrome, perineal fistula, presacral mass, recto‐ vestibular fistula, sacral hypoplasia dysgenesis, teratoma

## Abstract

Currarino Syndrome (CS) should be suspected in infants presenting with persistent constipation and sacral anomalies. Early diagnosis using appropriate imaging and multidisciplinary surgical management is essential to prevent serious complications such as bowel obstruction, infection, and neurological impairment.

## 1 Introduction

Currarino syndrome is a rare congenital malformation complex characterized by a distinctive constellation of developmental anomalies involving the caudal region of the body. It is traditionally defined by a triad consisting of anorectal malformations, sacral bony abnormalities, and the presence of a presacral mass. This syndrome represents a spectrum of anomalies rather than a single uniform condition, with significant variability in anatomical presentation and severity among affected individuals. The underlying embryological basis is thought to involve abnormal development of the caudal eminence during early gestation, leading to defects in the formation and separation of structures derived from the hindgut and caudal neural tube [1, 2, 3, 4]. Genetically, Currarino syndrome may occur sporadically or follow an autosomal dominant inheritance pattern, most commonly associated with mutations in the MNX1 (HLXB9) gene. These genetic alterations disrupt normal developmental signaling pathways, contributing to the phenotypic variability observed in affected individuals. Advances in molecular genetics have improved the understanding of this condition, although incomplete penetrance and variable expressivity continue to pose challenges in diagnosis and family counseling [1, 5, 6].

From a broader perspective, Currarino syndrome remains an important entity in pediatric surgical and developmental pathology due to its complex anatomical associations and potential long‐term functional implications. Early recognition and comprehensive evaluation are essential, as the condition often requires coordinated multidisciplinary management involving pediatric surgeons, radiologists, geneticists, and other specialists. Furthermore, understanding the spectrum of this syndrome contributes to improved diagnostic strategies, optimized treatment planning, and better long‐term outcomes.

This report aims to highlight the relevance of a structured and staged approach in the management of complex congenital anomalies associated with Currarino syndrome, emphasizing the importance of systematic evaluation and individualized care strategies in improving overall patient outcomes.

## 2 Case History and Examination

A female infant currently aged 11 months presented with progressive difficulty in stool passage since birth. Parents reported minimal stool passage from what was presumed to be an anus, with fecal discharge noted through the vaginal vestibule. The condition was associated with persistent constipation, progressive abdominal distension, poor weight gain, and recurrent perineal skin infection.

She was born at term via normal vaginal delivery with a birth weight of 2.9 kg and had an uneventful neonatal period. There was no known family history of anorectal malformations or sacral anomalies.

Physical examination revealed absence of a normally sited anal opening. The opening initially identified as the anus was found to be a perineal fistula, with an associated recto‐vestibular fistula, Figure 1. Contrast study via the perineal fistula confirmed features of a complex congenital Anorectal Malformation (ARM).

**FIGURE 1:**
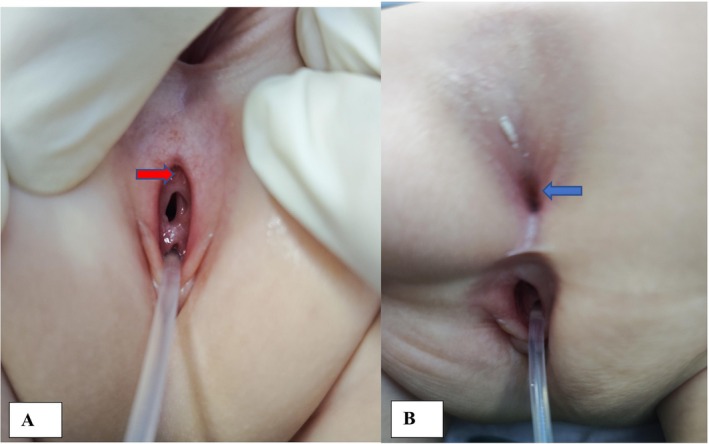
Vestibular fistula (red arrow) when the patient was in jackknife position in A and perineal fistula (blue arrow) in B.

Given the long‐standing constipation complicated by colitis, local infection, and poor perineal conditions, primary anoplasty and fistula repair were considered high risk. At 6 weeks of life, following one week of bowel preparation, a double‐barrel sigmoid colostomy was performed successfully, Figure 1. The postoperative course was uneventful, and the parents were educated on stoma care prior and the need for anoplasty before sigmoid colostomy closure.

## 3 Differential Diagnosis, Investigations and Treatment

Distal stoma contrast study demonstrated a patent distal bowel with contrast predominantly exiting through the recto‐vestibular fistula and a smaller amount via the perineal fistula. Figure 2. Owing to the triad of anorectal malformation, sacral abnormality, and presacral fullness, Magnetic Resonance Imaging (MRI) of the lumbosacral spine and pelvis was performed. This revealed a well‐defined fat‐rich presacral mass at the S3–coccygeal level measuring approximately 2.8 × 3.2 × 4.0 cm, causing anterior displacement and compression of the rectum, with partial sacral bone loss involving S3–S5 vertebrae, suggestive of a teratoma. Figure 3. Tumor markers (Alpha Feto Protein (AFP) and Beta Human Chorionic Gonadotrophin (β‐hCG)) were within age‐appropriate reference ranges. Genetic analysis identified a heterozygous MNX1 c.639G>A (p.Trp213Ter) variant, which was absent in both parents, consistent with a likely de novo mutation.

**FIGURE 2:**
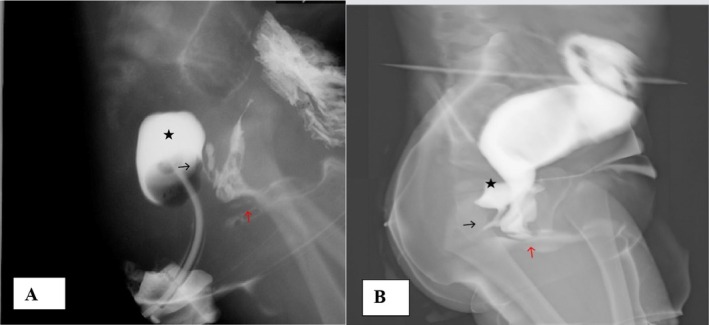
Distal colostogram with black arrow showing recto‐vestibular fistula, red arrow showing perineal fistula and five‐pointed star showing a folded rectum both in A and B.

**FIGURE 3:**
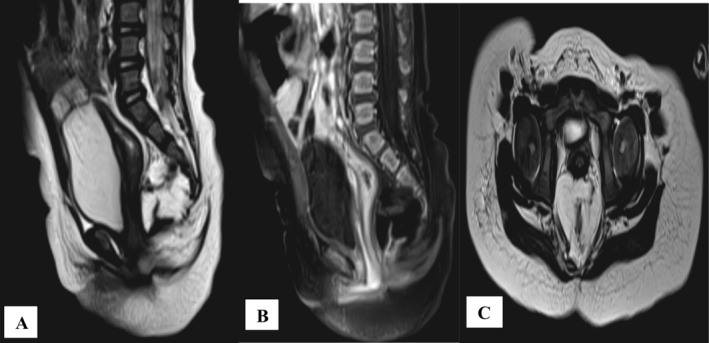
Magnetic resonance imaging (MRI) of the pelvis demonstrating the lesion. (A) Sagittal T2‐weighted MRI showing a well‐defined presacral cystic lesion (high T2 signal intensity) located posterior to the rectum and anterior to the sacrum, causing anterior displacement of the rectum without evidence of invasion into adjacent pelvic structures. (B) Sagittal contrast‐enhanced (T1‐weighted) MRI demonstrating the same presacral lesion with a smooth, well‐circumscribed wall and no appreciable solid enhancing component, supporting a benign cystic process. (C) Axial MRI showing the lesion occupying the presacral space in the midline, with preserved fat planes between the mass, rectum, and surrounding pelvic organs, without evidence of pelvic sidewall extension or adjacent organ invasion.

At the age of 8 months, the patient underwent definitive surgery Posterior Sagittal Anorectoplasty (PSARP) in which the original distribution of the anal sphincter was determined intra‐operatively using the neuromuscular electrical stimulator assisting in reconstruction of the normal neuromuscular structure of the anus Figure 4. It was revealed that the perineal fistula was communicating with the presacral cystic teratoma, explaining the recurrent perineal skin infections before colostomy. Therefore, en bloc excision of the presacral mass, anorectal reconstruction, and fistula repair with a multidisciplinary approach involving pediatric surgical and neurosurgical teams was conducted. Intra‐operatively, the neurosurgery team first exposed and repaired the dural defect through a limited posterior sacral exposure to prevent Cerebral Spinal Fluid (CSF) leak and meningitis; pediatric surgery then completed en bloc resection of the presacral mass via a posterior sagittal (presacral) approach and reconstructed a neo‐anus calibrated at 15 mm Figure 5. Estimated blood loss was minimal, and there were no intraoperative complications. Histopathology confirmed a benign mature teratoma with a microscopic section of a stained tissue revealing squamous epithelium, skin appendages, and fibrofatty tissue Figure 6. Postoperative recovery was uneventful, with improved bowel function, normal neurological development, and no evidence of recurrence on follow‐up labs and MRI. Three months post‐definitive surgery (at 11 months of age), the colostomy closure was done after achieving adequate neo‐anal size following serial anal dilatations Figure 7.

**FIGURE 4:**
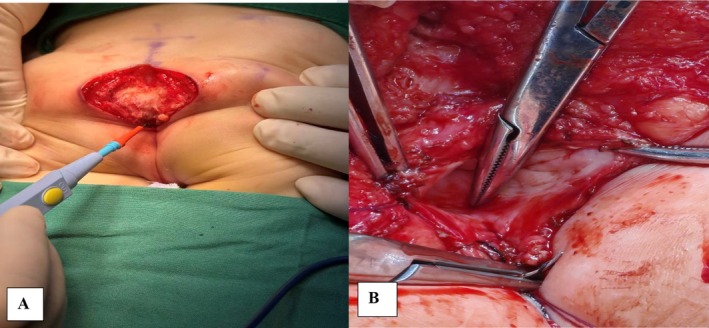
Start of the perineal dissection in A to demonstrate a perineal fistula opening communicated with presacral cyst which reached the S3 level and was tightly adhered to sacrum and coccyx and its wall was thick in B.

**FIGURE 5:**
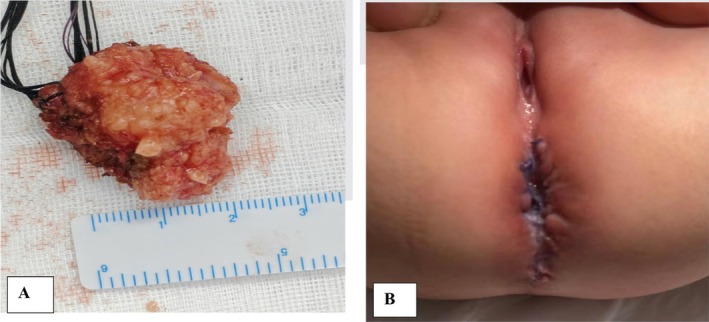
Presacral cyst (teratoma) after excision in A and reconstructed neo‐anus in B.

**FIGURE 6:**
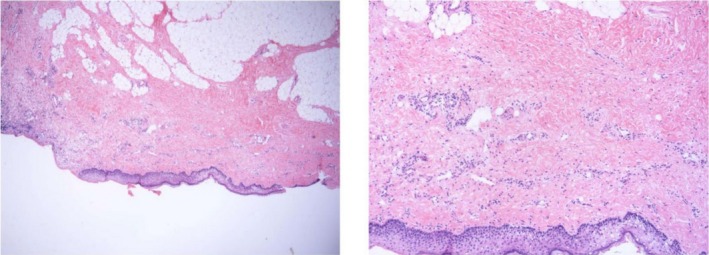
Histopathological examination of the excised lesion (hematoxylin and eosin stain). On the left is a low‐power view (**×**4 magnification) demonstrating keratinized stratified squamous epithelium overlying fibrofatty tissue with skin appendages, consistent with ectodermal differentiation. On the right is a higher‐power view (**×**20 magnification) showing mature fibrofatty tissue containing adnexal structures beneath the squamous epithelium without immature elements or cytological atypia, supporting the diagnosis of a mature cystic teratoma.

**FIGURE 7:**
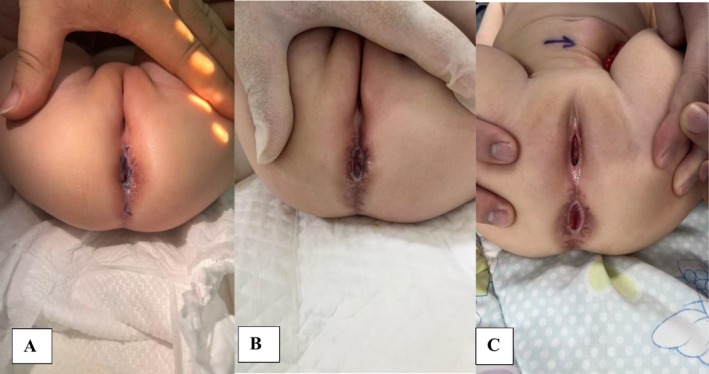
Neo‐anus at day 7 post PSARP in A, 2 months follow up before colostomy closure in B and on the day of colostomy closure in C.

## 4 Conclusion and Results

The findings confirm the classical triad of Currarino syndrome with a complex presacral component, highlighting the importance of detailed MRI evaluation and multidisciplinary planning. In this case, a staged surgical approach was deliberately adopted to optimize safety and functional outcomes. Initial fecal diversion allowed decompression of the distal bowel and minimized the risk of contamination during definitive repair. Subsequent posterior sagittal anorectoplasty (PSARP) enabled precise anatomical reconstruction of the anorectal malformation, while delaying colostomy closure ensured adequate healing and functional calibration of the neo‐anus.

This staged strategy is particularly advantageous in complex Currarino syndrome due to the coexistence of presacral masses and sacral dysraphism, which increase the risk of pelvic sepsis, neural injury, and repair breakdown if addressed in a single‐stage procedure. By separating the procedures, the approach reduces operative morbidity, allows better visualization and control of pelvic structures, and improves continence outcomes.

The favorable postoperative course in this patient with good stooling pattern, preserved neurologic function, and successful colostomy closure supports the staged approach as a safer and more effective management strategy in complex presentations of Currarino syndrome.

The patient had uneventful recovery following PSARP, with satisfactory perineal wound healing with well positioned neo‐anus. Three months later after a successful anal dilatation program, colostomy closure was performed without complications, with bowel functions returning to normal. At 6 months follow up, the baby was thriving, passing soft stool without constipation, incontinence, enterocolitis or anal stenosis, with perineal examination revealing well healed neo‐anus with good caliber. The patient remains under multidisciplinary follow up for long term assessment of bowel function, continence, and surveillance for other manifestation of Currarino syndrome.

## 5 Discussion

CS is a rare congenital disorder classically defined by the triad of ARM, sacral bony anomalies, and presacral masses [1, 7, 8]. Although the complete triad is considered pathognomonic, up to half of affected individuals present with incomplete or subtle manifestations, leading to delayed or missed diagnoses, sometimes into adolescence or adulthood [9, 10]. This phenotypic variability reflects the incomplete penetrance and variable expressivity of the underlying genetic defect, most commonly involving pathogenic variants of the MNX1 (HLXB9) gene [11, 12, 13].

Presacral masses are a central and clinically significant component of CS, occurring in approximately 60%–80% of cases [9, 10, 14]. Among these, anterior sacral meningoceles are the most frequent, followed by teratomas and dermoid or epidermoid cysts [7, 10, 15]. The intimate anatomical relationship between these lesions and the sacral canal explains the high risk of dural involvement and potential communication with the CSF space [7, 16, 17]. This relationship has important surgical implications, particularly with regard to the prevention of CSF leakage, meningitis, and long‐term neurological morbidity [7, 16, 17].

From a surgical perspective, excision of presacral masses in CS requires meticulous preoperative planning and multidisciplinary collaboration, especially between pediatric surgeons and neurosurgeons [7, 10, 18]. MRI plays a pivotal role in identifying intradural extension, anterior sacral meningoceles, tethered cord, or direct continuity between the presacral mass and the thecal sac [19, 20]. Failure to recognize these features preoperatively significantly increases the risk of intraoperative CSF leak [21, 22]. As the patient presented with severe and complex ARM (anal atresia, recto vestibular fistula, rectoperineal fistula) combined with presacral tumor and spinal lesions increased the surgical difficulty and risks, therefore necessitating multidisciplinary collaboration before and after surgery.

Neurosurgical involvement is particularly critical when a meningocele, dural defect, or tethered spinal cord is present [7, 20]. The primary neurosurgical objective during presacral mass excision is the identification and repair of any communication between the CSF space and the presacral compartment [7, 20]. This is achieved through watertight closure of the dura mater, either by primary suturing or, when necessary, by the use of autologous or synthetic dural grafts.

Watertight dural repair is the single most important factor in preventing postoperative CSF leak and its complications, including pseudomeningocele formation, wound breakdown, and meningitis [7, 20]. Adjunctive techniques such as reinforcement with fibrin sealants, multilayered closure using fascia and muscle flaps, and elimination of dead space further reduce leak risk [23, 24].

Regarding the surgical approach, posterior sagittal, transabdominal, or combined can be individualized based on tumor size, cranial extension, and relationship to pelvic organs and neural structures [10, 25]. Posterior sagittal approaches allow excellent exposure for simultaneous anorectal reconstruction and presacral mass excision in selected cases, but they also increase the likelihood of encountering dural abnormalities, reinforcing the need for neurosurgical expertise during these procedures [10, 25].

Beyond the immediate perioperative risks, long‐term surveillance is essential in CS. Although most presacral teratomas associated with CS are mature and benign, malignant transformation has been reported, particularly in older patients [15, 26, 27]. Histopathological findings therefore guide postoperative surveillance intensity, including serial imaging and tumor marker monitoring where appropriate [26].

In summary, Currarino syndrome represents a complex developmental disorder with significant surgical challenges. Presacral masses, particularly anterior sacral meningoceles and teratomas, place patients at high risk of CSF leak during surgical excision. Careful preoperative imaging, planned neurosurgical involvement, and meticulous watertight dural repair are essential to minimize complications and optimize outcomes. Early diagnosis and coordinated multidisciplinary management remain the cornerstones of successful treatment and long‐term prognosis in patients with Currarino syndrome.

## Author Contributions

**Ferdinand Medard Shilikale:** conceptualization, data curation, formal analysis, investigation, methodology, software, validation, visualization, writing – original draft, writing – review and editing. **Cui Mingyu:** conceptualization, data curation, investigation, methodology, validation, visualization, writing – review and editing. **Liu Guihai:** conceptualization, data curation, formal analysis, investigation, methodology, validation, visualization, writing – review and editing. **Rajabu Athumani Bakari:** conceptualization, data curation, formal analysis, investigation, methodology, software, supervision, validation, visualization, writing – original draft, writing – review and editing. **Jiao Chenwei:** conceptualization, data curation, formal analysis, investigation, methodology, supervision, validation, visualization, writing – review and editing.

## Funding

The authors have nothing to report.

## Ethics Statement

One uncommon situation that arose during our regular surgical procedures is the subject of this case report. This case report was therefore exempt from our institution's review board's ethical approval process.

## Consent

Written informed consent was obtained from the patient's parent for publication of this case report and any accompanying images. A copy of the written consent is available for review by the Editor‐in‐Chief of this journal on request.

## Conflicts of Interest

The authors declare no conflicts of interest.

## Data Availability

The corresponding author can provide the data supporting the study's conclusions upon request. Due to ethical and privacy concerns, the data are not publicly accessible.
